# ATP-driven conformational dynamics reveal hidden intermediates in a heterodimeric ABC transporter

**DOI:** 10.7554/eLife.110967

**Published:** 2026-07-31

**Authors:** Matija Pečak, Christoph Nocker, Robert Tampé

**Affiliations:** 1 Institute of Biochemistry, Biocenter, Goethe University Frankfurt Frankfurt Germany; University of Georgia United States; Stanford University United States

**Keywords:** ABC transporters, conformational dynamics, membrane proteins, single-molecule analysis, FRET, Förster resonance energy transfer, *E. coli*

## Abstract

ATP-binding cassette (ABC) transporters are essential molecular machines whose conformational dynamics have largely been inferred from ensemble-averaged measurements. Resolving dynamic heterogeneity and transient intermediates, however, requires single-molecule approaches. Here, we use single-molecule Förster resonance energy transfer (smFRET) to resolve ATP-driven conformational dynamics of the heterodimeric type IV ABC transporter TmrAB, a functional homolog of the human antigen transporter TAP, at the level of individual molecules. Fluorophores positioned at the nucleotide-binding domains and periplasmic gate were validated by accessible-volume simulations, fluorescence lifetimes, and ensemble FRET, demonstrating that these reporters reliably track conformational transitions. Single-molecule analysis distinguishes ATP-free and ATP-bound states and quantifies ATP-dependent population shifts from nucleotide-free to physiological ATP concentrations. Kinetic analysis further reveals an unexpectedly long ATP-bound dwell time of ~300 ms. Using complementary stabilization strategies, we directly resolve a previously hidden outward-facing open state that is kinetically masked under turnover conditions. These results provide the first single-molecule characterization of TmrAB and establish a quantitative single-molecule framework for dissecting ATP-coupled conformational dynamics in heterodimeric ABC transporters.

## Introduction

ATP-binding cassette (ABC) transporters constitute the largest family of primary active membrane transport systems, conserved across all domains of life (Davidson et al., 2008; Rees et al., 2009; Thomas and Tampé, 2020). Despite considerable structural diversity, all ABC transporters share a modular architecture comprising two conserved nucleotide-binding domains (NBDs)—the defining hallmark of the family—and two transmembrane domains (TMDs) that form the substrate translocation pathway (Thomas and Tampé, 2020; Locher, 2016). Based on their TMD architecture, ABC transporters are classified into seven types that encompass importers, exporters, extractors, and mechanotransmitters (Thomas et al., 2020). Substrate translocation is driven by large conformational changes that are chemo-mechanically coupled to ATP binding, hydrolysis, and phosphate/ADP release (Rees et al., 2009; Thomas and Tampé, 2020). ABC transporters play central roles in cellular homeostasis, nutrient uptake, waste removal, and toxin defense. Their dysfunction and misregulation are linked to numerous diseases and drug resistance (Robey et al., 2018).

The heterodimeric type IV ABC transporter TmrAB from *Thermus thermophilus* has emerged as a powerful model system due to its exceptional thermal stability and functional homology to the transporter associated with antigen processing (TAP1/2), a key component of adaptive immunity (Abele and Tampé, 2004; Kim et al., 2015; Zutz et al., 2011). Notably, TmrAB shares overlapping peptide specificity with TAP and can restore antigen presentation in TAP-deficient human cells (Nöll et al., 2017). Its inherent asymmetry, with one catalytically active (canonical) and one inactive (noncanonical) nucleotide-binding site (NBS), provides a unique opportunity to investigate functional specialization and asymmetry in ABC transport mechanisms.

Extensive structural studies, particularly using cryogenic electron microscopy (cryo-EM), have delineated the conformational landscape of TmrAB and yielded a detailed model of its translocation cycle (Hofmann et al., 2019; Stefan et al., 2020). In this model, TmrAB fluctuates between inward-facing wide and narrow conformations (IF^wide^ and IF^narrow^), characterized by a sealed periplasmic gate (PG) and well-separated NBDs, thereby permitting substrate access to the central binding cavity. ATP binding to both NBDs induces NBD dimerization and drives the transition into the outward-facing (OF) states, including an OF open (OF^open^) conformation with an open PG that enables substrate release into the periplasm, as well as an OF occluded (OF^occluded^) state characterized by a sealed PG and dimerized NBDs. Subsequent ATP hydrolysis and phosphate release lead to asymmetric unlocked return states (UR^asym^ and UR^asym^*), before the transporter returns to the IF conformation. These UR states feature a sealed PG, a partially open ADP-bound canonical NBS, and a tightly ATP-occluded noncanonical NBS (Hofmann et al., 2019).

Single-turnover experiments have shown that ATP binding, rather than hydrolysis, drives the IF-to-OF transition, while phosphate release precedes the OF-to-IF switch (Stefan et al., 2020; Stefan et al., 2021; Nocker et al., 2026). Complementary ensemble approaches, including pulsed electron–electron double resonance (PELDOR/DEER) spectroscopy, have further characterized ATP-dependent conformational changes (Barth et al., 2020; Barth et al., 2018). However, ensemble averaging inherently masks molecular heterogeneity, obscures inactive or misfolded subpopulations, and limits access to kinetic information.

Single-molecule techniques overcome these limitations by resolving conformational dynamics at the level of individual molecules (Agam et al., 2023; Hellenkamp et al., 2018). In particular, single-molecule Förster resonance energy transfer (smFRET) enables real-time monitoring of protein conformational changes with nanometer precision (Sasmal et al., 2016; Bartels et al., 2021; Lerner et al., 2021; Nettels et al., 2024). Applied to ABC transporters, smFRET provides a unique opportunity to dissect transport cycles, resolve transient intermediates, and extract kinetic and mechanistic insights that remain inaccessible to ensemble-based measurement approaches (Wang et al., 2020; Levring et al., 2023; Husada et al., 2018).

Here, we apply total internal reflection fluorescence (TIRF) microscopy combined with alternating laser excitation (ALEX)-based smFRET to detergent-solubilized heterodimeric ABC transporter TmrAB, providing the first single-molecule characterization of this system. By strategically positioning fluorophore pairs, we directly monitor ATP-dependent NBD dimerization and PG opening, quantify conformational state occupancies across ATP concentrations ranging from nucleotide-free to physiological levels (3 mM), and uncover conformational dynamics previously masked by ensemble averaging. Using three orthogonal trapping strategies—(i) a slow-turnover mutant (Hofmann et al., 2019; Stefan et al., 2020), (ii) Mg²^+^ depletion (Nocker et al., 2026; Barth et al., 2020), and (iii) substrate trans-inhibition (Grossmann et al., 2014; Sun et al., 2025)—we resolved a previously hidden outward-facing open (OF^open^) state that rapidly exchanges with the outward-facing occluded (OF^occluded^) state. Distance measurements derived from smFRET closely matched predictions from accessible-volume (AV) simulations, cryo-EM structures, and PELDOR/DEER spectroscopy, confirming that detergent-solubilized TmrAB retains a native-like conformational landscape. Together, these results provide the first single-molecule quantification of conformational state occupancies for a heterodimeric type IV ABC transporter and establish TmrAB as a versatile model for single-molecule studies of ABC transport systems.

## Results

### Design of FRET-labeled TmrAB variants to probe conformational dynamics

To monitor conformational changes in distinct regions of TmrAB, we engineered double-cysteine variants for smFRET targeting the nucleotide-binding domains (NBDs) and the PG. The NBDs undergo ATP-dependent dimerization and post-hydrolysis dissociation, whereas the PG opening and closing controls substrate release into the periplasm (Rees et al., 2009; Thomas and Tampé, 2020; Hofmann et al., 2019). Probing both regions provides complementary readouts of cytosolic and periplasmic coupling during transport.

Labeling positions were selected based on prior PELDOR/DEER studies (Barth et al., 2018). The NBD reporter variant (TmrA^C416^B^L458C^, referred to as TmrAB^NBD^) monitors the noncanonical NBS, while the PG reporter (TmrA^C416A, T61C^B^R56C^, hereafter TmrAB^PG^) reports PG opening. In TmrAB^NBD^, the native single cysteine (C416) was retained for labeling, whereas in TmrAB^PG^ it was substituted by alanine to prevent off-target labeling. Using the noncanonical NBS prevents direct distinction between OF^occluded^ and asymmetric unlocked return states (UR^asym^ and UR^asym^*) (Hofmann et al., 2019), reducing the number of resolvable FRET states and simplifying data interpretation.

**Figure 1.**
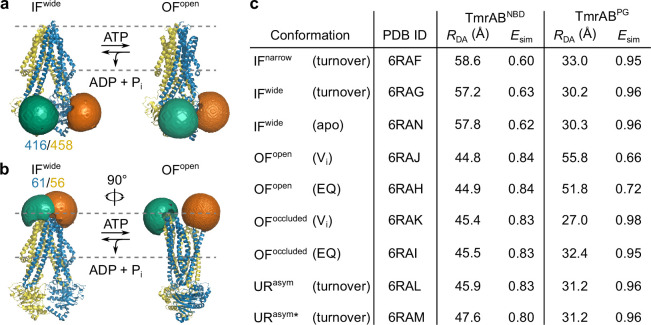
Accessible-volume (AV) simulations of LD fluorophores on TmrAB variants. AV simulations were performed for LD555 (donor) and LD655 (acceptor) fluorophores attached to the selected TmrAB labeling sites to assess whether donor–acceptor distances are suitable for single-molecule Förster resonance energy transfer (smFRET) measurements (Kalinin et al., 2012). (**a**) TmrAB^NBD^ (TmrA^C416^B^L458C^) and (**b**) TmrAB^PG^ (TmrA^C416A, T61C^B^R56C^) in the inward-facing wide (IF^wide^; PDB: 6RAN, left) and outward-facing open (OF^open^; PDB: 6RAH, right) conformations. The approximate membrane position is indicated by the dashed gray line. TmrA is shown in blue with LD655 (orange) and TmrB in yellow with LD555 (green), with labeling positions indicated (416/458 for TmrAB^NBD^; 61/56 for TmrAB^PG^). For illustration, fluorophores are shown in a defined configuration; however, in experiments, donor and acceptor dyes are stochastically attached, resulting in random distribution between subunits. Figure 1—source data 1.Excel file containing the data for the accessible-volumen (AV) simulation for Figure 1.

Both variants were labeled with photostable LD555/LD655 fluorophores containing a 1,3,5,7-cyclooctatetraene moiety to suppress photobleaching and blinking (Altman et al., 2011; Martin et al., 2023). AV simulations (Kalinin et al., 2012) across nine cryo-EM structures (Hofmann et al., 2019) confirmed that donor–acceptor distances (*R*_DA_) and simulated FRET efficiencies (*E*_sim_) lie within the measurable range (Figure 1 and Table 1). For TmrAB^NBD^, *E*_sim_ shifts from 0.62±0.02 (57.9±0.7 Å, NBDs separated) to 0.84±0.01 (45.2±0.4 Å, NBDs dimerized), corresponding to predicted Δ*E*_sim_ of 0.22±0.02 and Δ*R*_DA_ of 12.7±0.8 Å. For TmrAB^PG^, *E*_sim_ changes from 0.96±0.02 (30.6±0.4 Å, closed PG) to 0.69±0.03 (53.8±2.0 Å, open PG), corresponding to predicted Δ*E*_sim_ of 0.27±0.04 and Δ*R*_DA_ of 23.2±2.0 Å.

**Figure 1—figure supplement 1.**
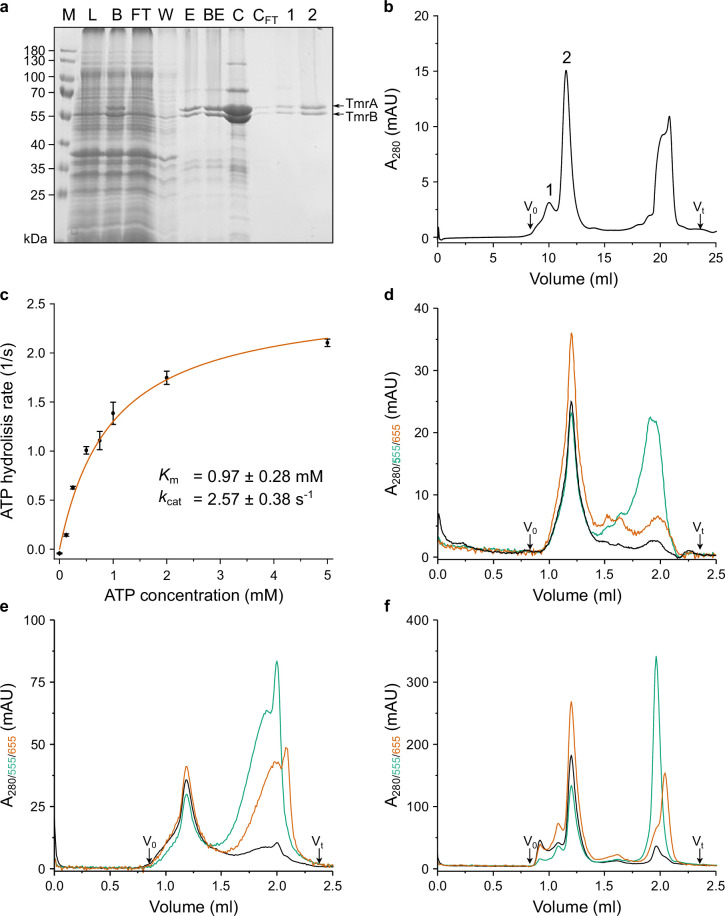
Quality of TmrAB purification and fluorophore labeling. (**a**) SDS-PAGE analysis (10%, reducing gel, Coomassie staining) of successive purification steps: M, molecular weight marker; L, cell lysate; B, Ni-NTA beads after incubation with lysate; FT, flow-through; W, wash; E, eluted TmrAB; BE, buffer-exchanged sample; C, concentrated protein; CFT, concentrator flow-through; 1 and 2, first and second peaks eluted from size-exclusion chromatography (SEC). Only the second peak was used for subsequent Förster resonance energy transfer (FRET) experiments. (**b**) SEC (Superdex 200 increase 10/300 GL) showing monodisperse labeled TmrAB and efficient removal of free fluorophores. A representative chromatogram of TmrAB^PG^ is shown. (**c**) ATP hydrolysis activity of purified wild-type TmrAB (60 nM TmrAB^wt^) measured at 40°C for 7 min using the Malachite Green assay. Released inorganic phosphate (P_i_) was quantified and data fitted to Michaelis–Menten kinetics, yielding *K*_m_ = 0.97 ± 0.28 mM and *k*_cat_ = 2.57 ± 0.38 s^–1^. (**d–f**) Analytical SEC (Superdex 200 increase 3.2/300) used to determine fluorophore labeling efficiencies of (**d**) TmrAB^NBD^, (**e**) TmrAB^PG^, and (**f**) TmrAB^PG_EQ^. LD555 and LD655 labeling efficiencies were ~55% and ~53% (TmrAB^NBD^), ~43% and ~52% (TmrAB^PG^), and ~42% and ~52% (TmrAB^PG_EQ^), respectively. Total cysteine occupancy exceeds 90% in all variants, reflecting near-complete dual-labeling with equimolar fluorophores. Figure 1—figure supplement 1—source data 1.Excel file containing the data for Figure 1–figure supplement 1. Figure 1—figure supplement 1—source data 2.Orignal SDS-PAGE image. Figure 1—figure supplement 1—source data 3.Orignal SDS-PAGE image, labeled.

**Figure 1—figure supplement 2.**
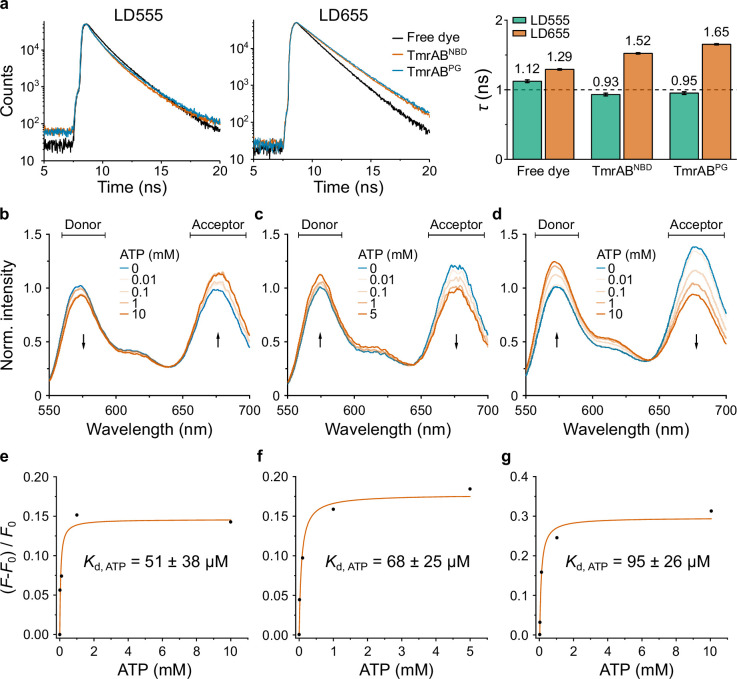
Förster resonance energy transfer (FRET) properties of labeled TmrAB variants. (**a**) Time-correlated single-photon counting histograms of LD555 (left) and LD655 (middle) measured for free dye in buffer (black), LD555/LD655-labeled TmrAB^NBD^ (orange), and LD555/LD655-labeled TmrAB^PG^ (blue). Amplitude-weighted average fluorescence lifetimes (*τ*) are summarized in the histogram (right), confirming sufficient fluorophore mobility for reliable FRET measurements. (**b–d**) Donor-excited ensemble emission spectra (550–700 nm, excitation 520 nm) of stochastic LD555/LD655-labeled (**b**) TmrAB^NBD^, (**c**) TmrAB^PG^, and (**d**) the slow-turnover variant TmrAB^PG_EQ^ measured at increasing ATP concentrations. Spectra were normalized to donor intensity in the apo state. ATP-dependent donor quenching and acceptor sensitization demonstrate that all variants retain FRET capability. (**e–g**) Fractional fluorescence changes, (*F-F*_0_)/*F*_0_, plotted as a function of ATP concentration for (**e**) TmrAB^NBD^, (**f**) TmrAB^PG^, and (**g**) TmrAB^PG_EQ^, where *F* is the acceptor emission intensity and *F*_0_ the intensity in the apo state. Data were fitted with a hyperbolic (Langmuir-type) binding model to determine apparent *K*_d_,_ATP_ values, which are consistent with ensemble FRET measurements of ATP binding. Figure 1—figure supplement 2—source data 1.Excel file containing the source data 1 for Figure 1–figure supplement 2. Figure 1—figure supplement 2—source data 2.Excel file containing the source data 2 for Figure 1–figure supplement 2.

**Figure 1—figure supplement 3.**
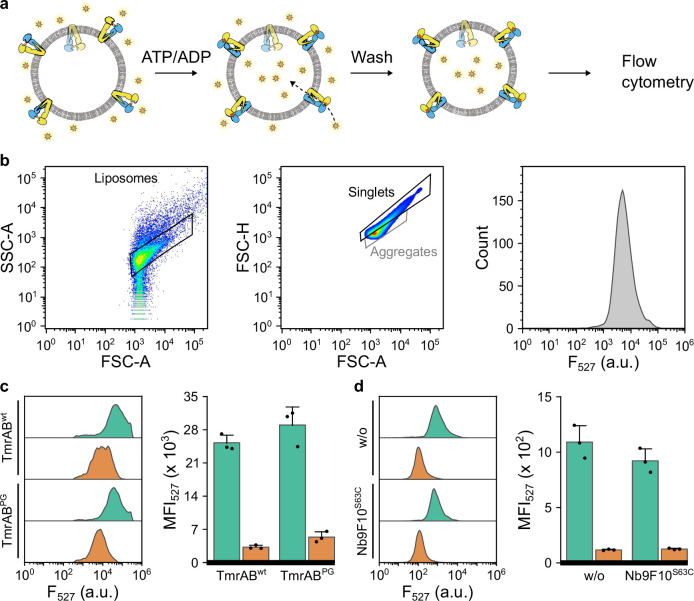
Effect of fluorophore labeling and nanobody binding on TmrAB transport activity. Single-liposome transport assays were performed by flow cytometry using mean fluorescence intensity at 527 nm (MFI_527_) as a readout of peptide transport. (**a**) TmrAB (0.6 µM) reconstituted into liposomes (~50 transporters per liposome) was incubated with C4F peptide (30 µM; RRYC^F^KSTEL, ^F^, fluorescein) in the presence of 3 mM ATP (green) or 3 mM ADP (orange) at 40°C for 5 min. After extensive washing, proteoliposomes were analyzed by flow cytometry. (**b**) Flow cytometry gating strategy. Forward scatter area (FSC-A) versus side scatter area (SSC-A; left) was used to identify homogeneous proteoliposomes, forward scatter height (FSC-H) versus FSC-A (middle) to select singlets, and fluorescence intensity at 527 nm (F_527_; right) to determine the mean fluorescence intensity (MFI) values of the gated population. (**c**) Transport activity of unlabeled wild-type TmrAB (TmrAB^WT^) compared with LD555/LD655-labeled TmrAB^PG^. (**d**) Effect of nanobody binding on TmrAB^wt^ transport activity. Assays were performed in the absence or presence of Nb9F10^S63C^ at a 1:1 molar ratio (0.6 µM each). Fluorophore labeling at the periplasmic gate and nanobody binding did not measurably affect TmrAB transport activity. Figure 1—figure supplement 3—source data 1.Excel file containing the source data 1 for Figure 1–figure supplement 3.

**Table 1.** Accessible-volume (AV) simulations of LD fluorophores on TmrAB variants. AV simulations were performed for LD555 (donor) and LD655 (acceptor) fluorophores attached to the selected TmrAB labeling sites to assess whether donor–acceptor distances are suitable for single-molecule Förster resonance energy transfer (smFRET) measurements (Kalinin et al., 2012). AV simulations confirm that donor–acceptor distances (*R*_DA_) remain within the FRET-sensitive range in both conformations, predicting measurable changes in simulated FRET efficiencies (*E*_sim_). Cryogenic electron microscopy (cryo-EM) structures of TmrAB reconstituted in lipid nanodiscs (Hofmann et al., 2019) were used as templates, including apo and ATP-bound (3 mM ATP) states. Outward-facing open (OF^open^) and outward-facing occluded (OF^occluded^) structures were obtained via orthovanadate trapping (V_i_) or the slow-turnover mutant TmrA^E523Q^B (EQ). Table 1—source data 1.Excel file containing the source data for Table 1.

			TmrAB^NBD^		TmrAB^PG^	
Conformation		PDB ID	RDA (Å)	*E*_sim_	RDA (Å)	*E*_sim_
IF^narrow^	(turnover)	6RAF	58.6	0.60	33.0	0.95
IF^wide^	(turnover)	6RAG	57.2	0.63	30.2	0.96
IF^wide^	(apo)	6RAN	57.8	0.62	30.3	0.96
OF^open^	(V_i_)	6RAJ	44.8	0.84	55.8	0.66
OF^open^	(EQ)	6RAH	44.9	0.84	51.8	0.72
OF^occluded^	(V_i_)	6RAK	45.4	0.83	27.0	0.98
OF^occluded^	(EQ)	6RAI	45.5	0.83	32.4	0.95
UR^asym^	(turnover)	6RAL	45.9	0.83	31.2	0.96
UR^asym^*	(turnover)	6RAM	47.6	0.80	31.2	0.96

A slow-turnover TmrAB variant (TmrA^C416A, E523Q, T61C^B^R56C^, hereafter TmrAB^PG_EQ^) was also generated to kinetically stabilize ATP-bound conformations. This mutation reduces ATP hydrolysis by ~1000-fold, extending the catalytic half-life of ~25 min at 45°C (Hofmann et al., 2019; Stefan et al., 2020; Nocker et al., 2026; Barth et al., 2020).

### TmrAB variants are suitable for FRET studies

TmrAB variants were expressed in *Escherichia coli* and purified by immobilized metal-affinity chromatography. SDS-PAGE and size-exclusion chromatography (SEC) confirmed purity and monodispersity of the samples (Figure 1—figure supplement 1a and b). ATPase assays showed that TmrAB^wt^ retained enzymatic activity after purification, yielding a Michaelis–Menten constant (*K*_m_) of 0.97±0.28 mM and turnover rate (*k*_cat_) of 2.57±0.38 s^–1^ at 40°C (Figure 1—figure supplement 1c), consistent with previous reports (Zutz et al., 2011; Nöll et al., 2017; Stefan et al., 2020).

Site-specific labeling yielded >90% labeling efficiency per cysteine, with ~40–50% per-site labeling efficiency for donor-only and acceptor-only populations (Figure 1—figure supplement 1d–f). Stochastic labeling of the TmrAB variants results in both homo (donor–donor, acceptor–acceptor) and hetero (donor–acceptor) labeled species. Only complexes with appropriate donor–acceptor stoichiometry were included in subsequent analyses.

Fluorescence lifetime (*τ*) analysis confirmed that the conjugated fluorophores retained their photophysical properties and sufficient rotational freedom for reliable FRET measurements. *τ* histograms of both conjugated and free fluorophores were fitted using a biexponential decay model, from which amplitude-weighted average lifetimes were calculated. For TmrAB^NBD^, average *τ* values were 0.93±0.02 ns (LD555) and 1.52±0.01 ns (LD655), whereas TmrAB^PG^ exhibited average *τ* values of 0.95±0.02 ns (LD555) and 1.65±0.01 ns (LD655) (Figure 1—figure supplement 2a). By comparison, free dyes in buffer displayed lifetimes of 1.12±0.02 ns (LD555) and 1.29±0.01 ns (LD655). The similarity between free and conjugated dye lifetimes, together with comparable values across variants, indicates minimal protein-induced quenching and supports sufficient orientational averaging for quantitative smFRET analysis (Hellenkamp et al., 2018; Ha and Tinnefeld, 2012; Sindbert et al., 2011; Dale et al., 1979; van der Meer, 2002).

Ensemble ATP titration (0–10 mM ATP) showed ATP-dependent donor quenching and acceptor sensitization (Figure 1—figure supplement 2b–d). ATP-induced fluorescence changes provided a quantitative readout of conformational transitions and enabled estimation of apparent equilibrium dissociation constants for ATP binding (*K*_d, ATP_) in labeled TmrAB variants (Figure 1—figure supplement 2e–g). The measured apparent *K*_d_,_ATP_ values were 51±38 μM for TmrAB^NBD^, 68±25 μM for TmrAB^PG^, and 95±26 μM for the slow-turnover variant TmrAB^PG_EQ^, consistent with prior biochemical measurements (~100 µM for TmrA^E523Q^B) (Stefan et al., 2020). Labeling did not measurably perturb ATP binding.

Previous studies on spin-labeled TmrAB^NBD^ demonstrated transport activity comparable to wild-type TmrAB (Barth et al., 2018), while AV simulations confirmed that fluorophores at these positions do not interfere with ATP- or substrate-binding sites. For the labeled TmrAB^PG^ variant, no previous transport activity had been reported; we therefore performed transport assays (Figure 1—figure supplement 3), which confirmed that LD555/LD655 labeling at the PG does not impair function, as labeled and wild-type TmrAB showed indistinguishable substrate transport in single-liposome assays (Figure 1—figure supplement 3c).

### ATP-induced conformational switching resolved by smFRET

**Figure 2.**
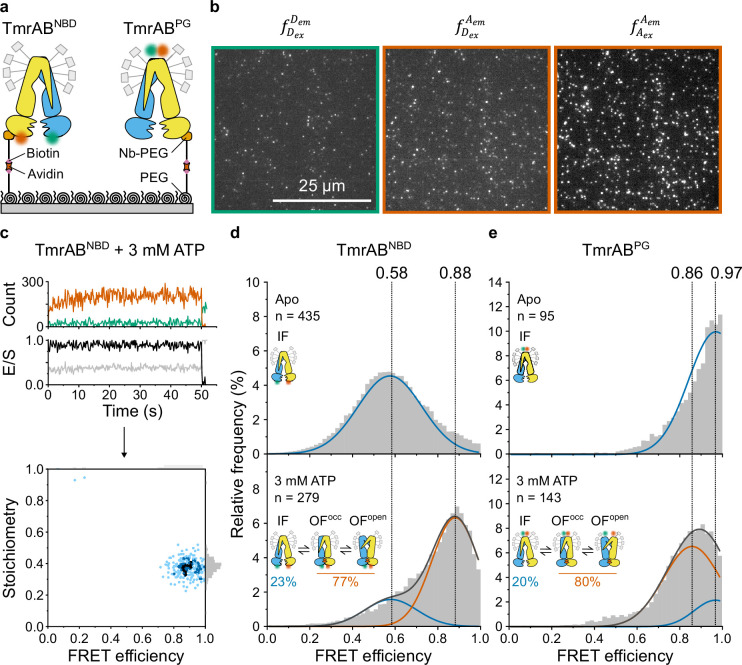
ATP-induced conformational changes of TmrAB analyzed by single-molecule Förster resonance energy transfer (smFRET). (**a**) Experimental setup. TmrAB^NBD^ (left) and TmrAB^PG^ (right) were labeled with LD555/LD655 and immobilized on PEGylated coverslips via a biotinylated conformation-independent, TmrB-specific nanobody (Nb9F10^S63C^) (Hofmann et al., 2019; Nocker et al., 2026). (**b**) smFRET imaging was performed using total internal reflection fluorescence (TIRF) microscopy with alternating laser excitation (ALEX; donor: 532 nm; acceptor: 640 nm). Emission was collected in donor (498–620 nm) and acceptor (662–710 nm) channels. Donor emission upon donor excitation ($f_{D_{ex}}^{D_{em}}$), sensitized acceptor emission upon donor excitation ($f_{D_{ex}}^{A_{em}}$), and direct acceptor emission upon acceptor excitation ($f_{A_{ex}}^{A_{em}}$) were used to calculate FRET efficiency (*E*) and stoichiometry (*S*). Data were analyzed using DeepFRET (Thomsen et al., 2020). (**c**) Representative TmrAB^NBD^ trace in ATP-bound state (top) and corresponding *E*/*S* plot (bottom). Donor emission upon donor excitation is shown in green (photon counts per frame), acceptor emission upon donor excitation in orange, FRET efficiency (*E*) in black, and stoichiometry (*S*) in gray. The initial increase in donor fluorescence reflects photophysical equilibration and instrumental stabilization and does not affect *E* or *S*, which are ratio-based and time-invariant (Hellenkamp et al., 2018). (**d, e**) Population analysis. FRET efficiency (*E*) histograms for (**d**) TmrAB^NBD^ and (**e**) TmrAB^PG^ are shown for apo (top) and ATP-bound (bottom; 3 mM ATP) conditions. Histograms were fitted with two Gaussian populations corresponding to the apo state (blue; defined from apo measurements) and ATP-bound state (orange; defined from saturating ATP fits). Dotted vertical lines indicate mean *E* values of each population, and relative fractions (Gaussian areas) are summarized schematically in each panel. Figure 2—source data 1.Excel file containing the source data 1 for Figure 2.

TmrAB was immobilized on PEGylated surfaces using a conformation-independent, TmrB-specific nanobody (Nb9F10^S63C^) (Hofmann et al., 2019). Previous studies confirmed that Nb9F10^S63C^ does not perturb TmrAB transport (Hofmann et al., 2019; Nocker et al., 2026), consistent with single-liposome transport assays performed in this study (Figure 1—figure supplement 3d). For surface immobilization, Nb9F10^S63C^ was conjugated to maleimide-PEG_11_-biotin, serving as an anchor to attach TmrAB to surface-immobilized streptavidin (Figure 2a). Donor and acceptor photons were recorded by a TIRF microscope using ALEX (NanoImager, ONI, Oxford, UK) at 40°C (Figure 2b). Single-molecule localization, fluorescence-trajectory extraction, and background correction were performed using NanoImager software, followed by DeepFRET-based machine-learning trace classification and corrections for donor leakage, direct acceptor excitation, and differences in detection efficiency (Hellenkamp et al., 2018; Thomsen et al., 2020; Hohlbein et al., 2014; Figure 2—figure supplements 1 and 2). FRET efficiency (*E*) and stoichiometry (*S*) were calculated from the corrected fluorescence trajectories (see Methods, Equation 1 and Equation 2), and 2D *E* versus *S* diagrams were constructed to verify that only complexes with appropriate donor–acceptor stoichiometry (*S* ≈ 0.5) were included in the smFRET analysis (Figure 2c).

**Figure 2—figure supplement 1.**
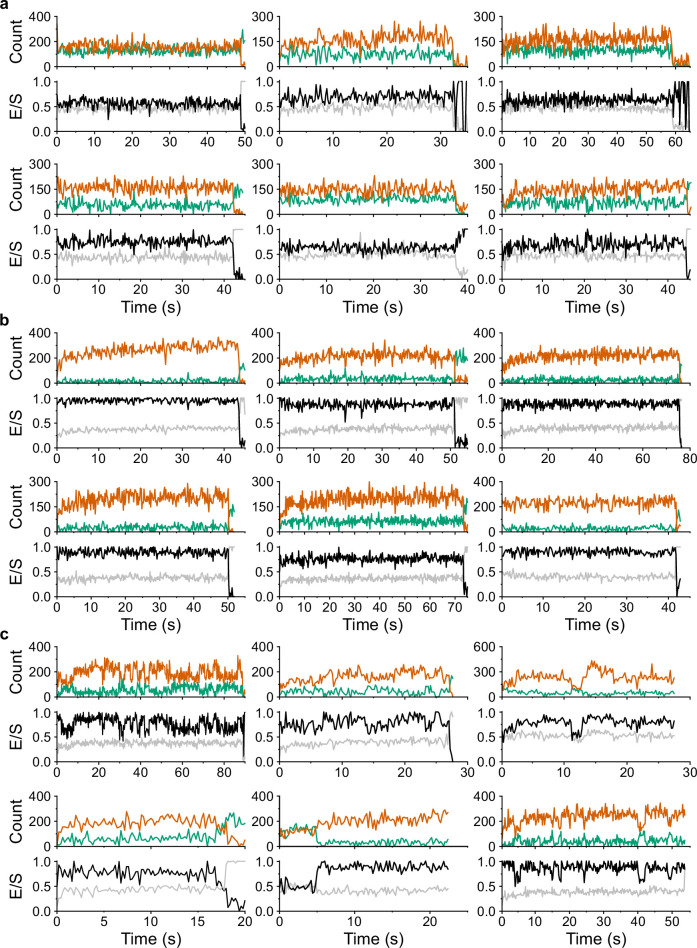
Representative single-molecule Förster resonance energy transfer (smFRET) traces of TmrAB^NBD^. Representative smFRET traces of TmrAB^NBD^ recorded (**a**) in the absence of ATP and (**b, c**) in the presence of 3 mM ATP. Hidden Markov modeling (HMM) classified ATP-bound traces as (**b**) static or (**c**) dynamic based on the absence or presence of transitions between ATP-free and ATP-bound conformational states. Donor fluorescence upon donor excitation is shown in green, acceptor fluorescence upon donor excitation in orange, FRET efficiency (*E*) in black, and stoichiometry (*S*) in gray. Figure 2—figure supplement 1—source data 1.Excel file containing the source data 1 for Figure 2–figure supplement 1. Figure 2—figure supplement 1—source data 2.Excel file containing the source data 2 for Figure 2–figure supplement 1.

**Figure 2—figure supplement 2.**
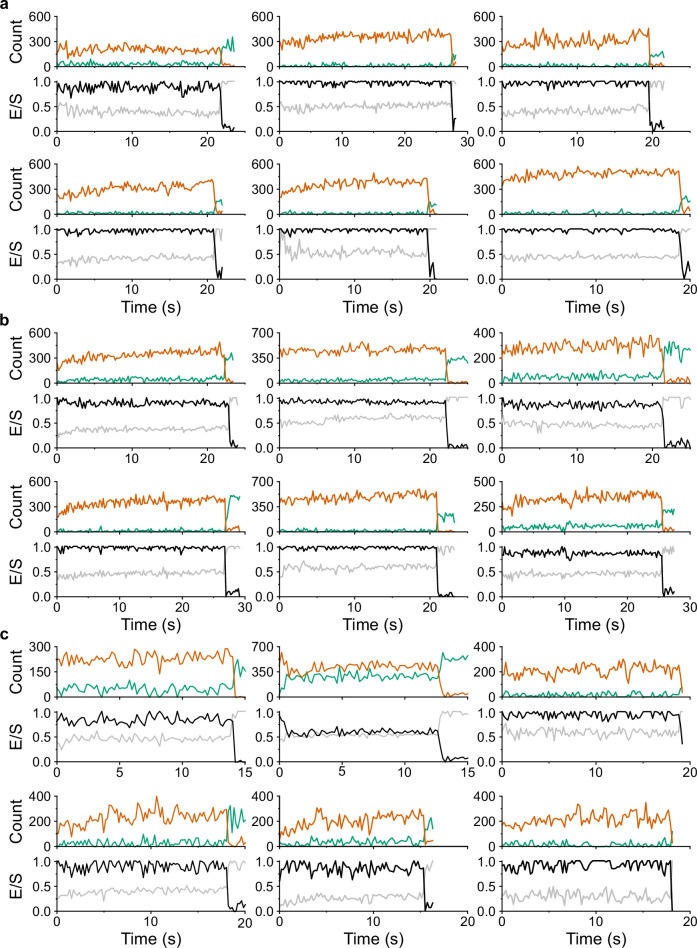
Representative single-molecule Förster resonance energy transfer (smFRET) traces of TmrAB^PG^. Representative smFRET traces of TmrAB^PG^ recorded (**a**) in the absence of ATP and (**b, c**) in the presence of 3 mM ATP. Hidden Markov modeling (HMM) classified ATP-bound traces as (**b**) static or (**c**) dynamic based on the absence or presence of transitions between ATP-free and ATP-bound conformational states. Donor fluorescence upon donor excitation is shown in green, acceptor fluorescence upon donor excitation in orange, FRET efficiency (*E*) in black, and stoichiometry (*S*) in gray.

FRET efficiency (*E*) histograms revealed two dominant Gaussian populations corresponding to apo and ATP-bound states (Figure 2d and e). Low-amplitude features were excluded from quantitative analysis because they were not reproducibly observed across replicates, lacked structural support from cryo-EM and DEER/PELDOR data, introduced poorly constrained fitting parameters, and increased the risk of overfitting. In the absence of ATP, only the apo population was observed, whereas addition of 3 mM ATP induced the appearance of a second ATP-bound population. For TmrAB^NBD^, the apo and ATP-bound populations exhibited mean *E* values of 0.58 and 0.88 (Δ*E*=0.30), respectively, with ~77% of molecules occupying the ATP-bound state. For TmrAB^PG^, mean *E* values were 0.97 (apo) and 0.86 (ATP-bound) (Δ*E*=0.11), with ~80% of molecules in the ATP-bound state.

Distance estimates using a Förster radius of *R*_0_=63.5 Å (Asher et al., 2021) yielded apparent distances of 60.2 Å (apo) and 45.6 Å (ATP-bound) for TmrAB^NBD^, and 35.6 Å (apo) and 46.9 Å (ATP-bound) for TmrAB^PG^. For TmrAB^NBD^, the experimentally derived Δ*R* of 14.6 Å closely agreed with the AV simulations. In contrast, the smaller Δ*R* of 11.4 Å observed for TmrAB^PG^ deviated from simulated values, suggesting that the ATP-bound population at this site represents a mixture of rapidly interconverting states rather than a single well-defined state.

### ATP dependence of conformational equilibria

**Figure 3.**
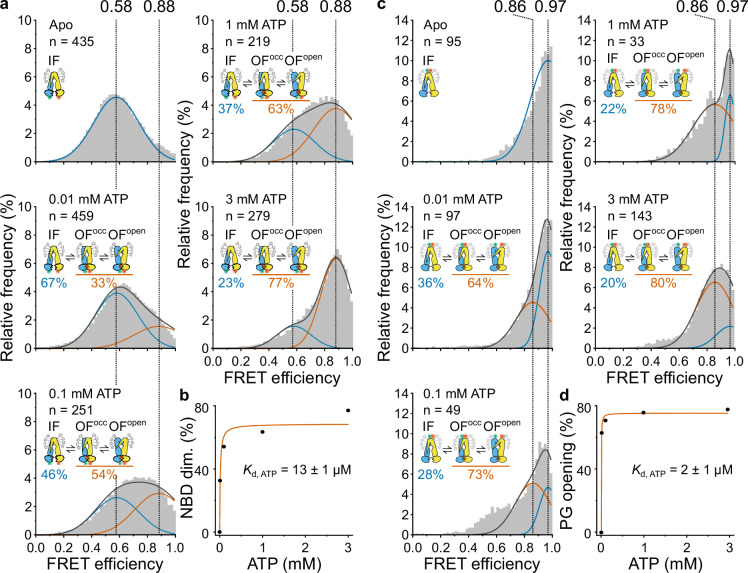
ATP-dependent shifts in single-molecule Förster resonance energy transfer (smFRET) populations of TmrAB. (**a, c**) Increasing ATP concentrations (0—3 mM) progressively shifted the population between apo and ATP-bound conformations for (**a**) TmrAB^NBD^ and (**c**) TmrAB^PG^. FRET efficiency (*E*) histograms were fitted with two Gaussian populations corresponding to the ATP-free state (blue; defined from apo samples) and the ATP-bound state (orange; determined from saturating ATP conditions). Dotted vertical lines indicate the mean *E* values of each state, and relative fractions (Gaussian areas) are summarized schematically in each panel. (**b, d**) ATP-binding curves obtained by plotting the fraction of molecules in the ATP-bound states as a function of ATP concentration for (**b**) TmrAB^NBD^ (reporting NBD dimerization) and (**d**) TmrAB^PG^ (reporting periplasmic gate [PG] opening). Data were fitted with a Langmuir isotherm to determine the apparent dissociation constant *K*_d, ATP_ of each variant. Figure 3—source data 1.Excel file containing the source data for Figure 3.

To assess the ATP sensitivity, we quantified conformational responses across a wide range of ATP concentrations, spanning well below the reported *K*_d, ATP_ (~100 µM for TmrA^E523Q^B) (Stefan et al., 2020) up to physiologically relevant levels (3 mM ATP). ATP concentrations up to 3 mM were selected to approximate near-physiological conditions commonly used in in vitro studies, where millimolar ATP levels ensure saturation of nucleotide-dependent conformational transitions and facilitate comparison with previous biochemical and structural analyses (Nöll et al., 2017; Hofmann et al., 2019; Stefan et al., 2020; Nocker et al., 2026). smFRET measurements revealed dose-dependent population shifts: TmrAB^NBD^ shifted from a low-FRET apo state (*E*=0.58) to a high-FRET ATP-bound state (*E*=0.88), whereas TmrAB^PG^ shifted from a high-FRET apo state (*E*=0.97) to a lower-FRET ATP-bound state (*E*=0.86) (Figure 3a and c). Langmuir isotherm fits yielded apparent *K*_d, ATP_ values of 13±1 μM for TmrAB^NBD^ and 2±1 μM for TmrAB^PG^ (Figure 3b and d), significantly lower than *K*_d ATP_ determined in ensemble FRET measurements (Figure 1—figure supplement 2e–g). This difference likely arises from the difficulty of deconvoluting overlapping FRET populations at sub-*K*_d, ATP_ concentrations, particularly for TmrAB^PG^, where state assignment is less well separated. Despite this quantitative offset, both approaches consistently indicate ATP saturation well below physiological concentrations and therefore support the same mechanistic conclusion that ATP binding drives conformational switching in TmrAB.

### Trapping of TmrAB^PG^ reveals a previously hidden OF open state

At 3 mM, ~80% of TmrAB^PG^ complexes populated the ATP-bound state (*E*=0.86), closely matching the ATP-bound population (~77% NBD-dimerized) observed for TmrAB^NBD^ (Figure 3). This agreement indicates that both labeling strategies consistently report ATP-dependent conformational changes. However, the ATP-induced shift observed for the periplasmic-gate reporter TmrAB^PG^ (Δ*E*=0.11, Δ*R*=11.4 Å; Figure 3c) was substantially smaller than predicted by AV simulations (Δ*E*=0.27, Δ*R*=23.2 Å; Table 1). This discrepancy suggests that the ATP-bound population at *E*=0.86 represents an unresolved ensemble, potentially comprising OF^open^ and OF^occluded^ conformations, as well as the post-hydrolysis asymmetric unlocked return states (UR^asym^ and UR^asym^*), which cannot be distinguished from OF^occluded^ within the current FRET geometry (Hofmann et al., 2019).

**Figure 4.**
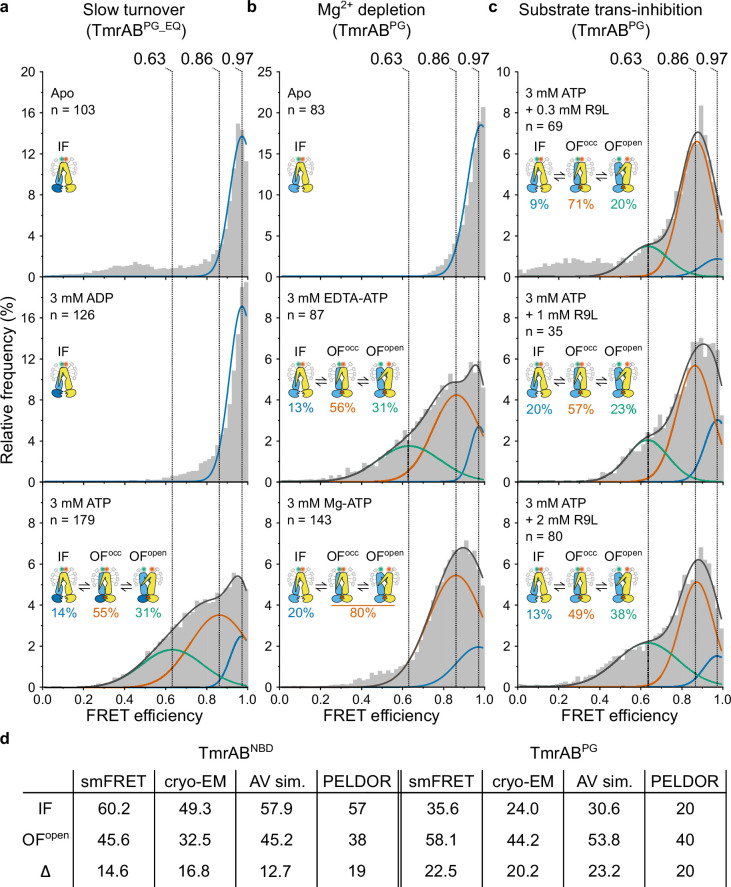
Identification of the outward-facing open (OF^open^) conformation. (**a–c**) Three complementary approaches were used to resolve the OF^open^ state: (**a**) the slow-turnover variant TmrAB^PG_EQ^, (**b**) imaging in Mg^2+^-free buffer supplemented with EDTA, and (**c**) stabilizing via trans-inhibition using high concentrations of peptide substrate R9L (0.3–2 mM). Förster resonance energy transfer (FRET) efficiency (E) histograms were fitted with three Gaussian populations corresponding to the ATP-free state (blue), the ATP-bound state (orange), and the OF^open^ state (green). All three strategies reveal a distinct OF^open^ population. Dotted vertical lines indicate the mean *E* values of each state, and relative fractions (Gaussian areas) are summarized schematically in each panel. (**d**) Comparison of inter-residue distances. Distances (Å) between selected residues on the nucleotide-binding domains (NBDs) and periplasmic gate (PG) of TmrAB (C_β_–C_β_) were determined using small-molecule FRET (smFRET) (this study, detergent-solubilized TmrAB), cryogenic electron microscopy (cryo-EM) structures of nanodisc-reconstituted TmrAB (PDB 6RAH, 6RAN) (Hofmann et al., 2019); accessible-volume (AV) simulations (this study), and pulsed electron–electron double resonance (PELDOR/DEER) measurements of detergent-solubilized TmrAB (Barth et al., 2018). Figure 4—source data 1.Excel file containing the source data for Figure 4.

To determine whether these states are kinetically unresolved by smFRET, we applied three complementary strategies to arrest the OF^open^ conformation of TmrAB^PG^: (i) a slow-turnover mutant (TmrAB^PG_EQ^), (ii) Mg²^+^ depletion using EDTA, and (iii) trans-inhibition by high concentrations of the substrate peptide R9L (RRYQKSTEL) (Figure 4). Slow-turnover variants have previously enabled structural separation of OF^open^ and OF^occluded^ states (Hofmann et al., 2019; Stefan et al., 2020; Nocker et al., 2026; Barth et al., 2020). Mg²^+^ depletion blocks ATP hydrolysis while preserving ATP binding, thereby enabling rapid and reversible trapping (Nocker et al., 2026). We further hypothesized that trans-inhibition by peptide binding sterically restricts PG closure and would therefore stabilize OF^open^ in a dose-dependent manner (Grossmann et al., 2014; Sun et al., 2025).

In the absence of ATP, either without nucleotide or in the presence of ADP (3 mM), the slow-turnover variant TmrAB^PG_EQ^ populated a single high-FRET state (*E*=0.97) (Figure 4a, top and middle). These results indicate that ADP binding alone is insufficient to promote either NBD dimerization or PG opening, which is consistent with previous biochemical and structural observations (Hofmann et al., 2019; Stefan et al., 2020). Upon ATP addition (3 mM), however, the conformational landscape diverged sharply from that of TmrAB^PG^. Instead of the two-state distribution observed for TmrAB^PG^ (apo: *E*=0.97, ~20%; ATP-bound: *E*=0.86, ~80%) (Figure 2e, bottom), TmrAB^PG_EQ^ exhibited three well-resolved populations with mean *E* values of 0.97 (~14%), *E*=0.86 (~55%), and *E*=0.63 (~31%) (Figure 4a, bottom).

As in TmrAB^PG^, the high-FRET population (*E*=0.97) corresponds to the IF state, whereas the intermediate-FRET population (*E*=0.86) likely represents a dynamic equilibrium between OF^open^ and OF^occluded^ conformations, as suggested by cryo-EM analyses (Hofmann et al., 2019). Post-hydrolysis return states (UR^asym^ and UR^asym^*) are expected to be minimally populated in TmrAB^PG_EQ^ because of its drastically reduced ATP hydrolysis rate (Stefan et al., 2020). Notably, the low-FRET ATP-bound population (*E*=0.63) was entirely absent in wild-type TmrAB^PG^. The transition from *E*=0.97 to *E*=0.63 corresponds to Δ*E*=0.34 and Δ*R*=22.5 Å, in close agreement with the IF→OF^open^ distance predicted by AV simulations (Δ*R*=23.2 Å; Figure 1 and Table 1), thereby supporting assignment of the *E*=0.63 population to the OF^open^ conformation.

Mg²^+^ depletion independently reproduced this three-state landscape. In the absence of Mg^2+^, wild-type TmrAB^PG^ transitioned from a single apo population (without ATP; *E*=0.97) (Figure 4b, top) to three ATP-bound populations (3 mM ATP; *E*=0.97, ~13%; *E*=0.86, ~56%; *E*=0.63, ~31%) (Figure 4b, middle), closely resembling those observed for the slow-turnover variant (Figure 4a, bottom). Reintroducing Mg²^+^ abolished the *E*=0.63 population and restored the wild-type TmrAB^PG^ two-state distribution (Figure 4b, bottom). This reversibility confirmed that the ATP-bound OF^open^ state (*E*=0.63) is selectively revealed only when ATP hydrolysis is prevented.

Finally, we tested whether periplasmic substrate binding shifts the conformational equilibrium of wild-type TmrAB^PG^ toward OF^open^. In the presence of ATP (3 mM), increasing concentrations of peptide substrate (0.3–2 mM R9L) progressively enriched the *E*=0.63 population from ~20% to ~38% (Figure 4c). This dose-dependent stabilization mirrors the trans-inhibition behavior reported for human TAP1/2 and reflects the upper substrate-loading capacity of the transporter (Grossmann et al., 2014).

Distance changes derived from smFRET closely match AV simulations, cryo-EM structures (PDB 6RAH, 6RAN) (Hofmann et al., 2019), and DEER/PELDOR measurements (Barth et al., 2020; Figure 4d), together validating assignment of the *E*=0.63 population as the OF^open^ conformation.

### Kinetics and thermodynamics of the transport cycle

ALEX-smFRET data were acquired with an effective temporal resolution of 200 ms (100 ms per excitation channel). Shorter integration times compromised the signal-to-noise ratio and precluded reliable FRET determination. To quantify conformational dynamics, we applied hidden Markov modeling (HMM) using MASH-FRET (Hadzic et al., 2018), classifying traces as either static (single FRET state) or dynamic (multiple states). Approximately 95% of traces in each condition were classified as static. The predominance of static traces is consistent with most conformational transitions occurring faster than the 200 ms observation window rather than reflecting long-lived conformational arrest.

Although individual transitions could not be directly resolved, population-based analysis (Figure 3), combined with biochemical turnover measurements (Figure 1—figure supplement 1c), allowed estimation of ATP-bound dwell times. Under saturating ATP conditions well above the apparent *K*_d, ATP_ (3 mM, 40°C), wild-type TmrAB exhibited a catalytic turnover rate of *k*_cat_ = 2.57 ± 0.38 s^–1^ (Figure 1—figure supplement 1c), corresponding to a full transport cycle time (*τ*_cycle_) of 395±55 ms. ATP-bound dwell times (*τ*_d_) were derived from population ratios obtained from Gaussian fits of the FRET efficiency histograms (Figure 3; see Methods, Equation 3 and Equation 4). These analyses yielded ATP-bound dwell times of 304±43 ms for TmrAB^NBD^ (~77% ATP-bound) and 316±44 ms for TmrAB^PG^ (~80% ATP-bound), with the remaining ATP-free intervals (~20–23%) accounting for 91 ms and 79 ms of the cycle, respectively.

The kinetic behavior of the ATP-bound ensemble is consistent with the thermodynamic properties previously determined for TmrAB. Although ATP-bound conformations accounted for most of the transport cycle (~77–80% occupancy), individual OF^open^ and OF^occluded^ states could not be resolved within the ATP-bound period (~310 ms), indicating rapid interconversion between these conformations. This observation agrees with previous thermodynamic analyses, showing that ATP binding drives the IF-to-OF transition with an overall free-energy change close to zero (Δ*G*=0.26 ± 0.74 kJ mol⁻¹), despite substantial compensating enthalpic and entropic contributions (Δ*H*=30.14 ± 2.80 kJ mol⁻¹; *T*Δ*S*=28.26 kJ mol⁻¹) (Barth et al., 2020). This entropy–enthalpy compensation places the conformational equilibrium near its midpoint, minimizing the energetic bias between ATP-bound conformations and thereby promoting rapid exchange between OF^open^ and OF^occluded^ states. Accordingly, the unresolved ATP-bound ensemble observed during steady-state turnover is consistent with a thermodynamic landscape that enables dynamic sampling of multiple OF conformations.

Together, these measurements establish a quantitative single-molecule description of both the kinetic progression and thermodynamic state occupancies throughout the catalytic cycle of a heterodimeric ABC transporter under active turnover conditions. By integrating population analysis with biochemical turnover measurements, this framework provides complementary insight into the dynamic and energetic landscape that governs the translocation cycle.

## Discussion

smFRET has become an indispensable tool for dissecting conformational dynamics of membrane proteins, including receptors, ion channels, and transporters, by directly linking structural transitions to functional states. With the ABC transporter family; however, smFRET studies have largely focused on monomeric or homodimeric systems (Wang et al., 2020; Levring et al., 2023; Husada et al., 2018), leaving asymmetric heterodimeric transporters comparatively underexplored. Here, we extend single-molecule analysis to the heterodimeric type IV ABC transporter TmrAB, revealing dynamic features of the transport cycle that are inaccessible to ensemble-averaged approaches.

By positioning FRET reporters at the NBDs and PG, we directly monitored ATP-dependent coupling between chemical energy input and global conformational rearrangements. Importantly, NBD dimerization is not uniquely coupled to a single OF state but can lead to either an OF^open^ or OF^occluded^ conformation (Hofmann et al., 2019; Stefan et al., 2020). This decoupling highlights the need to monitor both cytosolic and periplasmic regions to resolve the transport mechanism.

A limitation of the present study is the use of two FRET reporter pairs. While these positions were selected to probe key functional regions and validated by structural and spectroscopic benchmarks, additional labeling positions could in principle resolve further intermediates. However, increasing the number of states would also complicate quantitative analysis due to partially overlapping FRET populations, particularly for TmrAB^PG^. The current design therefore balances mechanistic resolution with robust state assignment.

Labeling sites previously validated for PELDOR/DEER spectroscopy (Nöll et al., 2017; Barth et al., 2020; Barth et al., 2018) were adapted for smFRET and rigorously benchmarked using AV simulations (Kalinin et al., 2012), fluorescence lifetime analysis, and ensemble FRET titrations. This validation is essential because fluorophores impose stricter steric and rotational constraints than spin labels (Sindbert et al., 2011). Collectively, these controls demonstrate that fluorophore attachment preserves native-like conformational behavior and ATP responsiveness. Fluorescence lifetime measurements further indicated efficient energy transfer without evidence of substantial protein-fluorophore quenching or restricted mobility (Hellenkamp et al., 2018).

Single-molecule measurements resolved two dominant FRET populations corresponding to apo and ATP-bound states for both TmrAB^NBD^ and TmrAB^PG^. ATP titrations from sub-*K*_d_,_ATP_ to physiological concentrations (3 mM ATP) produced gradual, concentration-dependent redistribution between these states, demonstrating high sensitivity of both reporters. Apparent *K*_d_,_ATP_ values derived from smFRET (2–13 µM) were lower than those obtained from ensemble FRET (50–100 µM). Importantly, both approaches agree that TmrAB reaches conformational saturation well below physiological ATP concentrations and retains Michaelis–Menten behavior.

Using three independent strategies—slow-turnover catalysis, Mg²^+^ depletion, and substrate trans-inhibition—we resolved this previously hidden OF^open^ conformation. The observed distance changes agree with cryo-EM structures (Hofmann et al., 2019), PELDOR/DEER data (Nöll et al., 2017; Barth et al., 2020; Barth et al., 2018), and AV-based predictions, indicating that detergent-solubilized TmrAB samples a largely native-like conformational landscape.

Although detergent and lipid nanodisc environments yield similar global states, fluorophore behavior may still be influenced by membrane proximity in lipid systems (Bartels et al., 2021; Dimura et al., 2016; Lam and Tajkhorshid, 2020). AV simulations suggest that dyes near the PG may experience steric constraints due to partial membrane overlap. While negligible under detergent conditions, this effect should be considered in future studies of membrane-reconstituted systems.

Substrate addition shifted the conformational equilibrium in a concentration-dependent manner, progressively stabilizing the OF^open^ state. This behavior is consistent with trans-inhibition observed in other ABC transporters, including TAP1/2 (Grossmann et al., 2014) and ABCC1 (Sun et al., 2025), and reflects finite substrate loading capacity (Grossmann et al., 2014).

**Figure 5.**
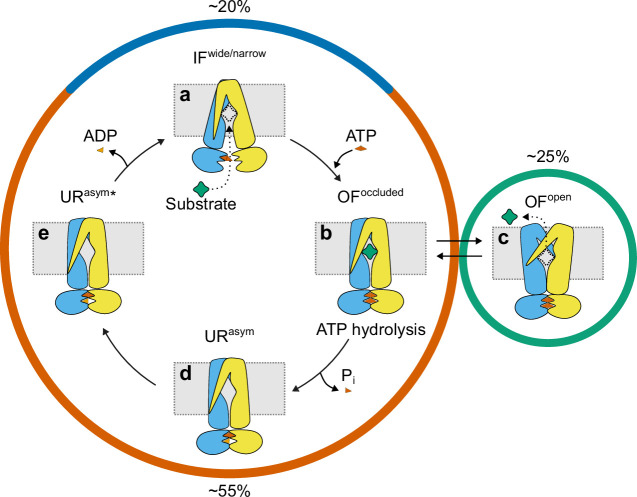
Conformational state distribution and catalytic cycle of TmrAB under active turnover. Schematic of the TmrAB transport cycle summarizing major conformational states and their estimated population distributions under physiological ATP concentrations (3 mM, 40°C). (**a**) The inward-facing apo state (IF^narrow^ and IF^wide^; blue arc) accounts for ~20% of molecules and is characterized by separated nucleotide-binding domains (NBDs) and a cytosol-accessible substrate-binding cavity. Substrate binding stabilizes the IF^wide^ conformation (Hofmann et al., 2019). ATP binding induces NBD dimerization and formation of the ATP-bound ensemble. (**b, c**) Under substrate-bound turnover conditions, TmrAB proceeds via (**b**) OF^occluded^ and (**c**) OF^open^ states in which substrate release occurs. In steady-state turnover, the ATP-bound ensemble rapidly interconverts between OF^occluded^ and OF^open^, accounting for ~25% of the ATP-bound population (green circle). These transitions occur faster than the ~200 ms temporal resolution of single-molecule Förster resonance energy transfer (smFRET) measurements, resulting in an averaged signal under turnover conditions. OF^occluded^ is proposed as an obligate intermediate between IF and OF^open^, preventing substrate backflow by maintaining an occluded binding cavity during rearrangements of the periplasmic gate (PG) and NBDs. Although a substrate-bound OF^occluded^ state has not been directly observed for TmrAB, its existence is supported by structures of the homodimeric type IV transporter BmrA (Chaptal et al., 2022). Reduced ATP hydrolysis or substrate trans-inhibition enables trapping of the OF^open^ state. (**d, e**) ATP hydrolysis and phosphate (P_i_) release generate post-hydrolysis return states (**d**) UR^asym^ and (**e**) UR^asym^*. Subsequent ADP release restores the apo IF conformation, completing the transport cycle. Overall, the ATP-bound phase (**b–e**) represents ~55% occupancy (orange arc) with an estimated dwell time of ~310 ms, whereas the apo/ATP-rebinding phase (**a**) lasts ~90 ms, yielding a total cycle time of ~400 ms (*k*_cat_ = 2.57 s^–1^). TmrA is shown in blue, TmrB in yellow, substrate as a green diamond, and nucleotides as orange symbols. Dotted gray boxes indicate the approximate position of the NBD dimer interface.

Quantitative deconvolution of FRET populations enabled estimation of conformational state occupancies under physiological ATP concentrations. During active turnover, TmrAB populates the IF state (~20%), OF^open^ (~25%), and OF^occluded^/post-hydrolysis states (UR^asym^ and UR^asym^*) (~55%; Figure 5). The current labeling scheme does not allow direct separation of OF^occluded^ from post-hydrolysis states due to fluorophore placement at the noncanonical NBS. However, post-hydrolysis contributions are expected to be reduced in the slow-turnover mutant TmrAB^PG_EQ^. To our knowledge, this represents the first single-molecule quantification of conformational equilibria in a heterodimeric ABC transporter under catalytic conditions. While IF substructure resolution is lower than in cryo-EM classifications (Hofmann et al., 2019; Nocker et al., 2026), smFRET uniquely captures ATP-bound intermediates that interconvert too rapidly for structural resolution.

All experiments were performed at 40°C for consistency with prior biochemical work and fluorophore stability. At the physiological temperature of *T. thermophilus* (68°C), absolute rates are expected to increase, although relative state distributions may be preserved if the free-energy landscape remains unchanged. Despite a substantial fraction of static trajectories, ATP-dependent shifts indicate that TmrAB operates near the temporal resolution of the measurements. Combining state occupancies with enzymatic turnover yields an ATP-bound dwell time of ~300 ms, consistent with previous biochemical estimates (Zutz et al., 2011; Stefan et al., 2020).

Emerging microsecond-resolution smFRET approaches could directly resolve short-lived intermediates (Grabenhorst et al., 2024), and multi-color FRET strategies (Bonhomme et al., 2025; Yim et al., 2012) may enable simultaneous monitoring of NBDs, PG, and nucleotide sites. These advances would allow direct detection of the OF^occluded^ states and post-hydrolysis dynamics.

Overall, the consistency between reporter positions, ensemble measurements, and structural data support the conclusion that the observed conformational states reflect native TmrAB behavior. While certain intermediates remain kinetically unresolved under turnover conditions, they are strongly supported by independent trapping approaches, reinforcing the robustness of the mechanistic model. Additional conditions, including nucleotide-state asymmetry and substrate-only binding, represent promising directions for future work.

In summary, this work establishes smFRET as a powerful approach for mapping the dynamic landscape of asymmetric ABC transporters. By quantitatively linking ATP binding, conformational equilibria, and kinetics at the single-molecule level, we define how chemical energy is converted into directional transport in heterodimeric ABC systems.

## Methods

**Key resources table:** 

Reagent type (species) or resource	Designation	Source or reference	Identifiers	Additional information
Strain, strain background (*Escherichia coli*)	BL21(DE3)	Thermo Fisher Scientific	Cat. Nr.: EC0114	Expression of His_10_-tagged TmrAB variants and nanobody Nb9F10^S63C^
Gene (*Thermus thermophilus*)	TmrAB^wt^	Hofmann et al., 2019	Plasmid ID: AT30	Wild-type TmrAB
Gene (*T. thermophilus*)	TmrAB^NBD^ (TmrA^C416^B^L458C^)	Barth et al., 2020	Plasmid ID: AT08	TmrAB variant
Gene (*T. thermophilus*)	TmrAB^PG^ (TmrA^C416A, T61C^B R^56C^)	Barth et al., 2020	Plasmid ID: AT27	TmrAB variant
Gene (*T. thermophilus*)	TmrAB^PG_EQ^ (TmrA^C416A, E523Q, T61C^B ^R56C^)	Barth et al., 2020	Plasmid ID: AT01	Slow-turnover TmrAB variant
Gene (*Vicugna pacos*)	Nb9F10^S63C^ (nanobody)	Hofmann et al., 2019	Plasmid ID: BH16	TmrB-specific, conformation-independent nanobody
Peptide, recombinant protein	R9L	This study	N/A	RRYQKSTEL
Peptide, recombinant protein	C4F	This study	N/A	RRYC^F^KSTEL; ^F^, fluorescein
Chemical compound, drug	LD555 (maleimide)	Lumidyne Technologies	CAT #4	FRET donor
Chemical compound, drug	LD655 (maleimide)	Lumidyne Technologies	CAT #8	FRET acceptor
Chemical compound, drug	ATP (adenosine 5'-triphosphate)	Sigma-Aldrich	CAS Nr.: 74804-12-9	
Chemical compound, drug	ADP (adenosine 5'-diphosphate)	Sigma-Aldrich	CAS Nr.: 20398-34-9	
Chemical compound, drug	EDTA (ethylenediaminetetraacetic acid)	Sigma-Aldrich	CAS Nr.: 6381-92-6	
Chemical compound, drug	*n*-Dodecyl β-D-maltoside (β-DDM)	Carl Roth	CAS Nr.: 69227-93-6	
Chemical compound, drug	Trolox	Sigma-Aldrich	CAS Nr.: 53188-07-1	
Chemical compound, drug	Streptavidin	Sigma-Aldrich	CAS Nr.: 9013-20-1	
Chemical compound, drug	Biotin-PEG_11_-maleimide	Sigma-Aldrich	Cat. Nr.: QBD10195	
Chemical compound, drug	*E. coli* polar lipids	Avanti Polar Lipids	CAS Nr.: 1240502-50-4	
Chemical compound, drug	Dioleoylphosphatidylcholine (DOPC)	Avanti Polar Lipids	CAS Nr.: 4235-95-4	
Commercial assay, kit	Ni-NTA Agarose	Bio-Rad	Cat. Nr.: 30250	IMAC purification of His_10_-tagged TmrAB
Software, algorithm	NanoImager software	ONI	165527-1aaf735	Microscope software; v1.19.16.20250510
Software, algorithm	DeepFRET	Thomsen et al., 2020		Machine-learning FRET trace classification; v2.0.5
Software, algorithm	MASH-FRET	Hadzic et al., 2018		Hidden Markov modeling of smFRET traces; v1.3.4
Software, algorithm	FluoFit	PicoQuant		Fluorescence decay analysis; v4.6.6 (discontinued)
Software, algorithm	OriginPro 2024	OriginLab		Data analysis; v10.1.0.178
Software, algorithm	FlowJo	Waters Biosciences	RRID:SCR_008520	Flow cytometry data analysis; v10.6.1
Software, algorithm	AV simulation toolkit	Kalinin et al., 2012	Zenodo: 3517287	Accessible-volume simulations

### Expression, purification, and labeling of TmrAB

His_10_-tagged TmrAB variants were expressed in *E. coli* BL21(DE3) (Thermo Fisher Scientific) as described previously (Stefan et al., 2020). Cells were grown in high-salt LB media (Carl Roth) supplemented with 100 μg ml^–1^ ampicillin (PAA Laboratories) at 37°C. At an OD_600_ of 0.5, expression was induced with 1 mM isopropyl β-D-thiogalactopyranoside (IPTG; Carl Roth), and cultures were incubated for 3 hr at 37°C. Cells were harvested by centrifugation (4500×*g*, 4°C, 15 min) and stored at –80°C.

For purification, cell pellets were resuspended in lysis buffer (20 mM HEPES-NaOH pH 7.5, 300 mM NaCl, 50 µg ml^–1^ lysozyme, 0.2 mM phenylmethylsulfonyl fluoride [PMSF]) and lysed by sonication. Cell debris was removed by centrifugation (18,000×*g*, 4°C, 35 min), and membranes were collected by ultracentrifugation (100,000×*g,* 4°C, 30 min). Membranes were solubilized for 2 hr at 4°C in purification buffer (20 mM HEPES-NaOH pH 7.5, 300 mM NaCl) containing 20 mM *n*-dodecyl β-D-maltoside (β-DDM; Carl Roth). After ultracentrifugation (100,000×*g,* 30 min, 4°C), the supernatant was incubated with Ni-NTA agarose (Bio-Rad) for 1 hr at 4°C. The resin was washed with 20 CV of wash buffer (20 mM HEPES-NaOH pH 7.5, 300 mM NaCl, 1 mM β-DDM) containing 50 mM imidazole, and TmrAB was eluted with elution buffer (20 mM HEPES-NaOH pH 7.5, 300 mM NaCl, 1 mM β-DDM, 300 mM imidazole).

For fluorophore labeling, TmrAB variants were conjugated via maleimide chemistry using LD555 and LD655 (Lumidyne Technologies). Labeling was carried out at a 1:10:10 molar ratio of protein to each dye in elution buffer for 3 hr at 4°C. Excess dye was quenched with 2 mM β-mercaptoethanol (Sigma-Aldrich), and the labeled protein was buffer-exchanged into SEC buffer (20 mM HEPES-NaOH pH 7.5, 150 mM NaCl, 1 mM β-DDM) using Zeba Spin Desalting Columns (Thermo Fisher Scientific). Unreacted fluorophores were removed by SEC on a Superdex 200 Increase 10/300 GL column (Cytiva). Labeling efficiency was determined by analytical SEC (Superdex 200 Increase 3.2/300; Cytiva) by monitoring absorbance at 280, 555, and 655 nm. To preserve sample integrity for smFRET measurements, TmrAB was purified and labeled within a single day, stored on ice, and imaged over the following 2 days.

### Time-correlated single-photon counting

Fluorescence lifetime measurements were performed using a FluoTime 100 spectrometer (PicoQuant) equipped for time-correlated single-photon counting (TCSPC). Experiments were carried out on labeled TmrAB in SEC buffer (20 mM HEPES-NaOH pH 7.5, 150 mM NaCl, 1 mM β-DDM). LD555 and LD655 were excited at 510 nm and 610 nm, respectively. Emission was collected using a 620/60 nm bandpass filter for LD555 and a BG4 700 nm long-pass filter for LD655. Photon arrival times were accumulated until the TCSPC histogram reached a peak count of 50,000 photons. Fluorescence decay curves were analyzed by fitting mono- or bi-exponential decay models using FluoFit software (PicoQuant), and the amplitude-weighted average of fluorescence lifetime was calculated.

### Nanobody production and purification

The nanobody Nb9F10^S63C^ was expressed and purified as described previously (Hofmann et al., 2019). Briefly, Nb9F10^S63C^ was produced in *E. coli* BL21(DE3) cells grown in Terrific Broth (TB; Carl Roth) supplemented with 100 μg ml^–1^ ampicillin at 37°C. At an OD_600_ of 0.6, expression was induced with 1 mM IPTG, followed by overnight incubation at 28°C. Cells were harvested by centrifugation (4500×*g*, 4°C, 15 min) and stored at –80°C. For purification, cell pellets were resuspended in nanobody lysis buffer (25 mM HEPES-NaOH pH 7.4, 300 mM NaCl, 15 mM imidazole, 0.5 mM PMSF) and disrupted by sonication. Cell debris was removed by centrifugation (18,000×*g*, 4°C, 35 min), and the clarified lysate was applied to Ni-NTA agarose equilibrated in potassium phosphate (KP_i_) buffer (25 mM KP_i_ pH 6.5, 100 mM KCl, and 0.5 mM tris(2-carboxyethyl) phosphine [TCEP]). Bound nanobody was washed with 10 column volumes (CV) of KP_i_ buffer and eluted with 8 CV of elution buffer (25 mM KP_i_ pH 6.0, 20 mM KCl, 300 mM imidazole, 0.5 mM TCEP). Eluted fractions were pooled and further purified by cation exchange chromatography on a HiTrap SP column (Cytiva) using a linear gradient from low-salt buffer (25 mM KP_i_ pH 6.0, 20 mM KCl, 0.5 mM TCEP) to high-salt buffer (25 mM KP_i_ pH 6.0, 500 mM KCl, 0.5 mM TCEP). The purified nanobody was concentrated and buffer-exchanged into nanobody SEC buffer (20 mM HEPES-NaOH pH 7.5, 150 mM NaCl) using Zeba Spin Desalting Columns, followed by SEC (Superdex 200 Increase 10/300 GL; Cytiva). For site-specific conjugation, Nb9F10^S63C^ was incubated with a biotin-PEG_11_-maleimide linker (Sigma-Aldrich) at a 1.2:1 molar ratio of protein to linker in the presence of 0.5 mM TCEP for 2 hr at 4°C. Excess linker was removed by desalting on Zeba Spin Desalting Columns, followed by a final SEC step (Superdex 200 Increase 10/300 GL).

### SDS-PAGE

The purity of TmrAB samples was assessed by SDS-PAGE. Resolving gels (10%) were prepared using 10% (wt/vol) acrylamide, 0.5 M Tris-HCl (pH 8.8), 0.13% (wt/vol) SDS, 0.05% (wt/vol) ammonium persulfate (APS), and 0.25% (vol/vol) *N*,*N*,*N’*,*N’-*tetramethylethylenediamine (TEMED). Stacking gels contained 4.3% (wt/vol) acrylamide, 0.5 M Tris/HCl (pH 6.8), 0.09% (wt/vol) SDS, 0.09% (wt/vol) APS, and 0.33% (vol/vol) TEMED. Gels were used immediately or stored at 4°C for up to 4 weeks. Protein samples were mixed with 4× SDS loading buffer containing dithiothreitol (Sigma-Aldrich) and heated at 90°C for 5 min before loading. Electrophoresis was performed at a constant voltage of 120 V using 1× SDS running buffer (25 mM Tris-HCl pH 8.8, 192 mM glycine, 0.1% SDS). Proteins were visualized by staining with InstantBlue Protein Stain (Abcam) for 1 hr at room temperature with gentle agitation and imaged using a Fusion FX system (Vilber).

### ATPase activity assay

The ATPase activity of β-DDM-solubilized TmrAB^wt^ was quantified using a Malachite Green-based colorimetric assay as described previously (Diederichs and Tampé, 2021). Detergent-solubilized TmrAB (60 nM) was incubated in ATPase buffer (20 mM HEPES-NaOH pH 7.5, 150 mM NaCl, 2 mM MgCl_2_, 1 mM β-DDM) containing 3 mM ATP (Sigma-Aldrich) at 40°C for 7 min. Autohydrolysis controls were prepared by incubation of ATP in ATPase buffer without protein. Reactions were quenched by adding 20 mM H_2_SO_4_, followed by incubation with 3 mM Malachite Green (Thermo Fisher Scientific), 0.2% (vol/vol) Tween20 (Carl Roth), and 1.5% (wt/vol) ammonium molybdate (Carl Roth) for 10 min at room temperature. The absorbance at 620 nm was recorded on a CLARIOstar v.5.20 R5 plate reader (BMG LABTECH).

### Ensemble FRET measurements

The ATP binding and FRET characteristics of selected TmrAB variants were assessed by ensemble FRET. Labeled TmrAB (100 nM) was incubated with increasing concentrations of ATP at 42°C for 5 min. Donor-excited emission was recorded from 550 to 700 nm with an excitation wavelength of 520 nm using a CLARIOstar v.5.20 R5 plate reader (BMG LABTECH). Acceptor emission intensities at 675 nm were plotted against ATP concentration and fitted with a hyperbolic function to determine the apparent dissociation constant (*K*_d, ATP_) for each variant.

### Reconstitution of TmrAB

TmrAB variants were reconstituted into proteoliposomes composed of *E. coli* polar lipids and dioleoylphosphatidylcholine at a molar ratio of 7:3. A protein-to-lipid ratio of 1:20 (wt/wt) was used, corresponding to approximately 50 TmrAB complexes per liposome. Extruded large unilamellar vesicles were destabilized by incubation with 0.3% (vol/vol) Triton X-100 for 30 min. TmrAB was then added to the detergent-destabilized liposomes and incubated for 30 min at 8°C under gentle rotation. Detergent was removed gradually using sequential addition of polystyrene Bio-Beads SM-2 (Bio-Rad): two incubations at 40 mg ml^–1^ (1 hr and overnight), followed by two incubations at 80 mg ml^–1^ (1 hr each). Proteoliposomes were subsequently harvested by ultracentrifugation (100,000×*g*, 30 min, 4°C) and resuspended in the appropriate buffer.

### Single liposome transport assay

Single liposome transport assay was performed as described previously (Stefan et al., 2020; Stefan et al., 2021). Wild-type TmrAB or LD555/LD655-labeled TmrAB^PG^ (0.6 µM each), reconstituted into liposomes (~50 TmrAB per liposome), was mixed with fluorescently labeled peptide RRYC^F^KSTEL (30 µM C4F; ^F^, fluorescein), 3 mM ATP or ADP, and 5 mM MgCl_2_ in liposome buffer (25 mM HEPES-NaOH pH 7.5, 150 mM NaCl, 5% [vol/vol] glycerol). Where indicated, nanobody Nb9F10^S63C^ (0.6 µM) was added. Samples were incubated for 5 min at 40°C (unless specified otherwise), followed by the addition of ice-cold ethylenediaminetetraacetic acid (EDTA; 10 mM). Proteoliposomes were then washed twice by centrifugation (270,000×*g*, 30 min) to remove non-transported peptides. Mean fluorescence intensities (MFIs) of translocated substrates were quantified by flow cytometry using a FACSMelody (Waters Biosciences) and analyzed in FlowJo 10.6.1 (Waters Biosciences). The gating strategy used to determine fluorescence intensities of single liposomes from raw flow cytometry data consisted of three steps: First, forward scatter area versus side scatter area (FSC-A vs SSC-A) was used to separate populations based on size and granularity. Second, forward scatter height versus forward scatter area (FSC-H vs FSC-A) was used to exclude aggregates and retain singlet events. Finally, fluorescence intensities of the gated singlet population were quantified at the wavelength of interest (527 nm), and the resulting values were used to calculate MFI of the proteoliposome population.

### Solid-phase peptide synthesis

The peptides used in this study were synthesized and labeled as described previously (Nocker et al., 2026; Löffler et al., 2024). The 9-mer peptides RRYQKSTEL (R9L) and RRYC^F^KSTEL (C4F; ^F^, fluorescein) were synthesized on preloaded Fmoc-L-Leu resin using a Liberty microwave-assisted peptide synthesizer (CEM) according to a standard protocol (54 W, 3 min, 75°C). Each coupling step was performed twice using 0.2 M fluorenylmethoxycarbonyl (Fmoc)-protected amino acids, 0.5 M O-benzotriazole-*N*,*N*,*N*’,*N*’-tetramethyluronium hexafluoro-phosphate (HBTU), and 1-hydroxybenzotriazole hydrate (HOBt H_2_O). Fmoc deprotection was carried out with 20% (vol/vol) piperidine and 0.1 M HOBt H_2_O in dimethylformamide (DMF). Peptides were cleaved from the resin using a cleavage cocktail containing 92.5% (vol/vol) trifluoroacetic acid (TFA), 2.5% (vol/vol) H_2_O, 4.5% (vol/vol) thioanisole, and 0.5% (vol/vol) 1,2-ethanedithiol (EDT) for 1.5 hr at room temperature. Cleaved peptides were precipitated in ice-cold diethyl ether (Et_2_O), pelleted, dissolved in tert-butanol (*t*BuOH)/water (4:1, vol/vol), and lyophilized. Peptides were purified by reverse-phase (RP) C18 HPLC (PerfectSil C18 column; MZ-Analysentechnik) using buffer A (Milli-Q water containing 0.05% [vol/vol] TFA) and buffer B (acetonitrile containing 0.05% [vol/vol] TFA). Peptide identity and purity were confirmed by LC-MS. For fluorescence labeling, the peptide RRYC^F^KSTEL was dissolved in 33% (vol/vol) DMF in PBS (10 mM Na_2_HPO_4_, 1.8 mM KH_2_PO_4_, 137 mM NaCl, 2.7 mM KCl, pH 7.4) and incubated for 1 hr at room temperature with a 1.3-fold molar excess of 5-iodoacetamido fluorescein (Merck).

### Functionalization of glass slides for single-molecule FRET analysis

Glass coverslips used for TmrAB immobilization in smFRET experiments were functionalized by PEGylation as described previously (Chandradoss et al., 2014). Coverslips (Carl Roth) were cleaned by sequential sonication in Milli-Q water and analytical-grade acetone (>99.9%; VWR International), followed by oxygen plasma treatment (0.3 mbar, 80% power, 15 min) using a Zepto plasma cleaner (Diener) and a 10 min incubation in methanol (Avantor, Gliwice, PL). Coverslips were then silanized by incubation for 30 min in a solution of 100 ml methanol, 5 ml acetic acid, and 3 ml 3-aminopropyltrimethoxysilane (APTES; Tokyo Chemical Industry). After silanization, slides were rinsed four times with methanol and dried under a nitrogen stream. Surface functionalization was achieved using a mixture of biotinylated-PEG (4 mol%) and nonbiotinylated-PEG (96 mol%; Rapp Polymer). The PEG solution was sandwiched between two coverslips and incubated overnight in a humidity chamber. Coverslips were then rinsed thoroughly with Milli-Q water and dried under nitrogen. To enhance passivation, a second PEGylation step was performed using 25 mM CH_3_-PEG-NHS (333 Da; Thermo Fisher Scientific) under the same conditions. Finally, slides were rinsed with Milli-Q water, dried under nitrogen, and stored at –20°C under argon until use.

### smFRET imaging

smFRET experiments were performed using a flow chamber system (Ibidi). Chambers were assembled by placing a biotin-PEG-functionalized glass slide onto a μ-Slide I Luer Family flow channel (Ibidi), with the functionalized surface facing inward. All buffers and Milli-Q water were filtered through 0.2 μm filters (Sigma-Aldrich). Chambers were washed with 1 ml Milli-Q water and incubated with 0.2 mg ml^–1^ streptavidin (Sigma-Aldrich) at 4°C for 30 min to allow binding to the biotin-PEG surface. Unbound streptavidin was removed by washing with 1 ml Milli-Q water. The surface was then treated with 0.3 mg ml^–1^ biotinylated-PEG_11_-Nb9F10^S63C^ at 4°C for 45 min, followed by flushing with 2 ml SEC buffer (20 mM HEPES-NaOH pH 7.5, 150 mM NaCl, 1 mM β-DDM). Detergent-solubilized, fluorophore-labeled TmrAB (100 nM) was added and incubated at 4°C for 1 hr. Unbound protein was removed by five washes with 1 ml TmrAB-SEC buffer. Chambers were equilibrated with 1 ml of imaging buffer containing 25 mM HEPES-NaOH (pH 7.5), 150 mM NaCl, 3 mM MgCl_2_, 50 mM glucose, 5 mM Trolox, 7.5 U ml^–1^ pyranose oxidase, and 1 kU ml^–1^ catalase, supplemented with the desired ATP concentration. For EDTA trapping experiments, MgCl_2_ was omitted and replaced with 3 mM ethylenediaminetetraacetic acid (EDTA; Sigma-Aldrich). smFRET data were acquired at 40°C using ALEX on a TIRF microscope (NanoImager S, ONI, Oxford, UK). Typically, 600 frames were recorded per region of interest with 100 ms exposure time. Laser powers were 0.8 mW cm^–2^ (532 nm) and 0.9 mW cm^–2^ (640 nm). Data were recorded in 1 min intervals, except for TmrAB^NBD^ variant in apo and 3 mM ATP conditions, where 3 min intervals were used to confirm that conformational transitions do not occur on timescales longer than 1 min due to the reduced temperature.

### smFRET data analysis

smFRET measurements were performed using ALEX, allowing assignment of detected photons based on both excitation and emission wavelengths. Photon counts for each molecule were extracted using NanoImager software (ONI NanoImager, Development Build) and classified into three detection channels: donor excitation with donor emission ($f_{D_{ex}}^{D_{em}}$), donor excitation with acceptor emission ($f_{D_{ex}}^{A_{em}}$), and acceptor excitation with acceptor emission ($f_{A_{ex}}^{A_{em}}$). Traces were analyzed with DeepFRET (Thomsen et al., 2020) and manually curated. To minimize bias, a second researcher independently curated traces from both ATP-free and 3 mM ATP samples, yielding 98% overlap between curations. FRET efficiency (*E*) and stoichiometry (*S*) were calculated as:

(1) $$\displaystyle E=\frac{f_{D_{ex}}^{A_{em}}}{f_{D_{ex}}^{D_{em}}+f_{A_{ex}}^{A_{em}}}$$

(2) $$\displaystyle S=\frac{f_{D_{ex}}^{D_{em}}+f_{D_{ex}}^{A_{em}}}{f_{D_{ex}}^{D_{em}}+\ f_{D_{ex}}^{A_{em}}+f_{A_{ex}}^{A_{em}}}$$

Population analysis was performed by constructing one-dimensional histograms of FRET efficiency (*E*) and stoichiometry (*S*) using OriginPro 2024 (OriginLab). Histograms were fitted with two Gaussian distributions corresponding to the ATP-free state (defined from apo samples) and the ATP-bound state (defined by a two-component fit at saturating ATP). HMM of individual traces was performed using MASH-FRET (Hadzic et al., 2018) to distinguish dynamic from static molecules within each sample.

### ATP-bound dwell times and distribution of conformational states

The ATP-bound dwell time (*τ*_d_) was estimated as:

(3) $$\displaystyle \tau _{d}=\tau _{cycle}\times f_{ATP-bound}$$

where *f*_ATP-bound_ is the fraction of ATP-bound molecules derived from Gaussian fits of the FRET efficiency histograms (Figure 3). This approach assumes (i) ATP hydrolysis occurs exclusively at the canonical NBS, with negligible contribution from the noncanonical site (Zutz et al., 2011; Hofmann et al., 2019; Barth et al., 2020), and (ii) the majority of molecules are catalytically competent and continuously cycling.

The distribution of conformational states within ATP-bound FRET population (*E*=0.86) of TmrAB^PG^ under turnover conditions (3 mM ATP) was determined using a two-state model:

(4) $$\displaystyle E=f_{open}\times E_{open}+f_{other}\times E_{other}$$

where *f*_open_ is the fraction of OF^open^ state (*E*_open_ = 0.63) readily resolved under trapping conditions (Figure 4), and *f*_other_ represents the combined fraction of PG-closed states (OF^occluded^/UR^asym^/UR^asym^*, *E*_other_ = 0.97).

## Data Availability

Source data are provided with this paper: DOI: https://doi.org/10.25716/gude.16a1-m6pe. The following dataset was generated: Goethe-Universität Frankfurt 2026ATP-driven conformational dynamics reveal hidden intermediates in a heterodimeric ABC transporterGoethe University Data Repository10.25716/gude.16a1-m6pePMC1342734342535643

## Additional files

MDAR checklist
