# Supplementary figures and images for: ATP-driven conformational dynamics reveal hidden intermediates in a heterodimeric ABC transporter
Source: eLife. 2026 Jul 31;15:RP110967. doi: 10.7554/eLife.110967 (PMC13427343; doi:10.7554/eLife.110967)

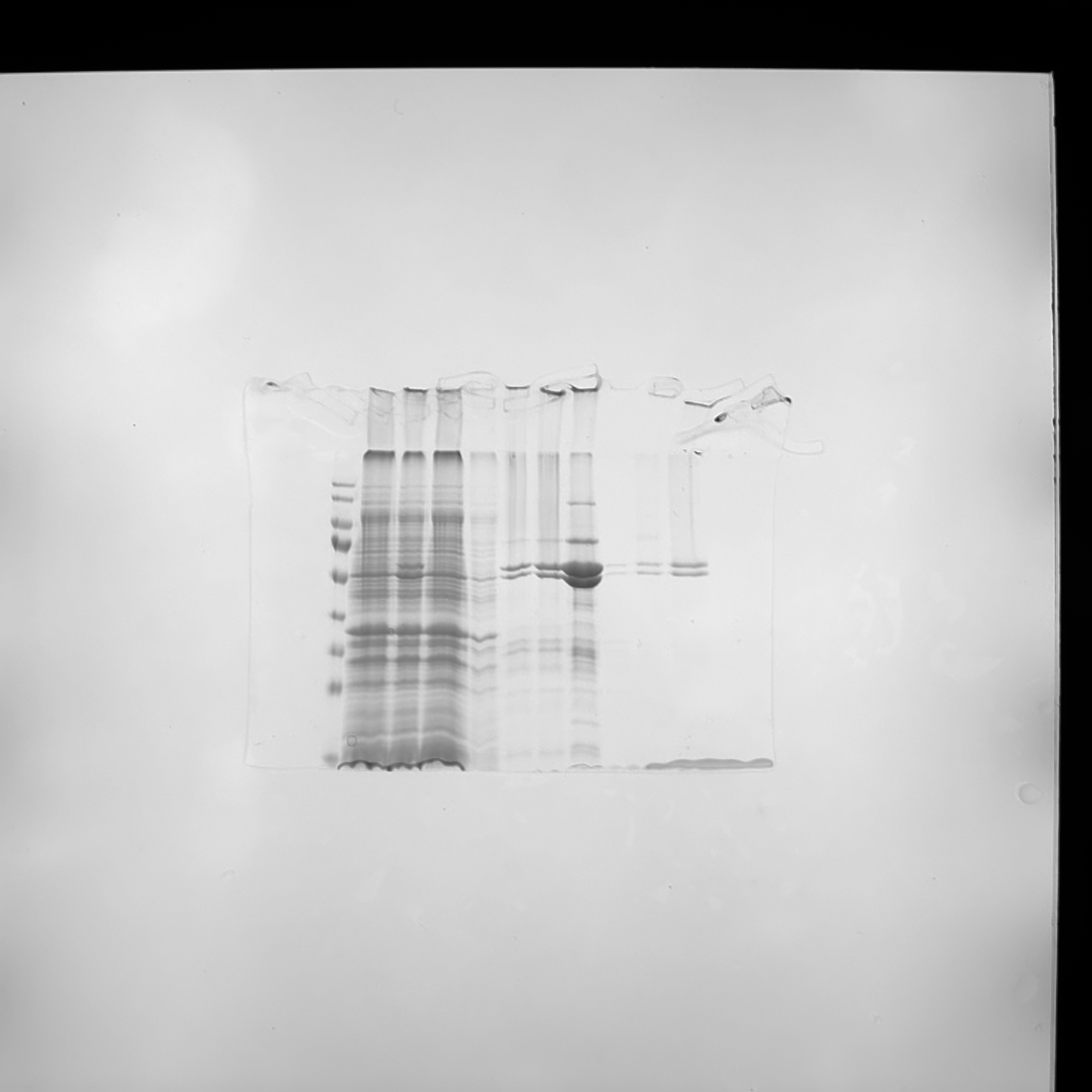

Supplement: Figure 1—figure supplement 1—source data 2. [file elife-110967-fig1-figsupp1-data2.zip › SDS-PAGE TmrAB purification_raw unedited gel.png]

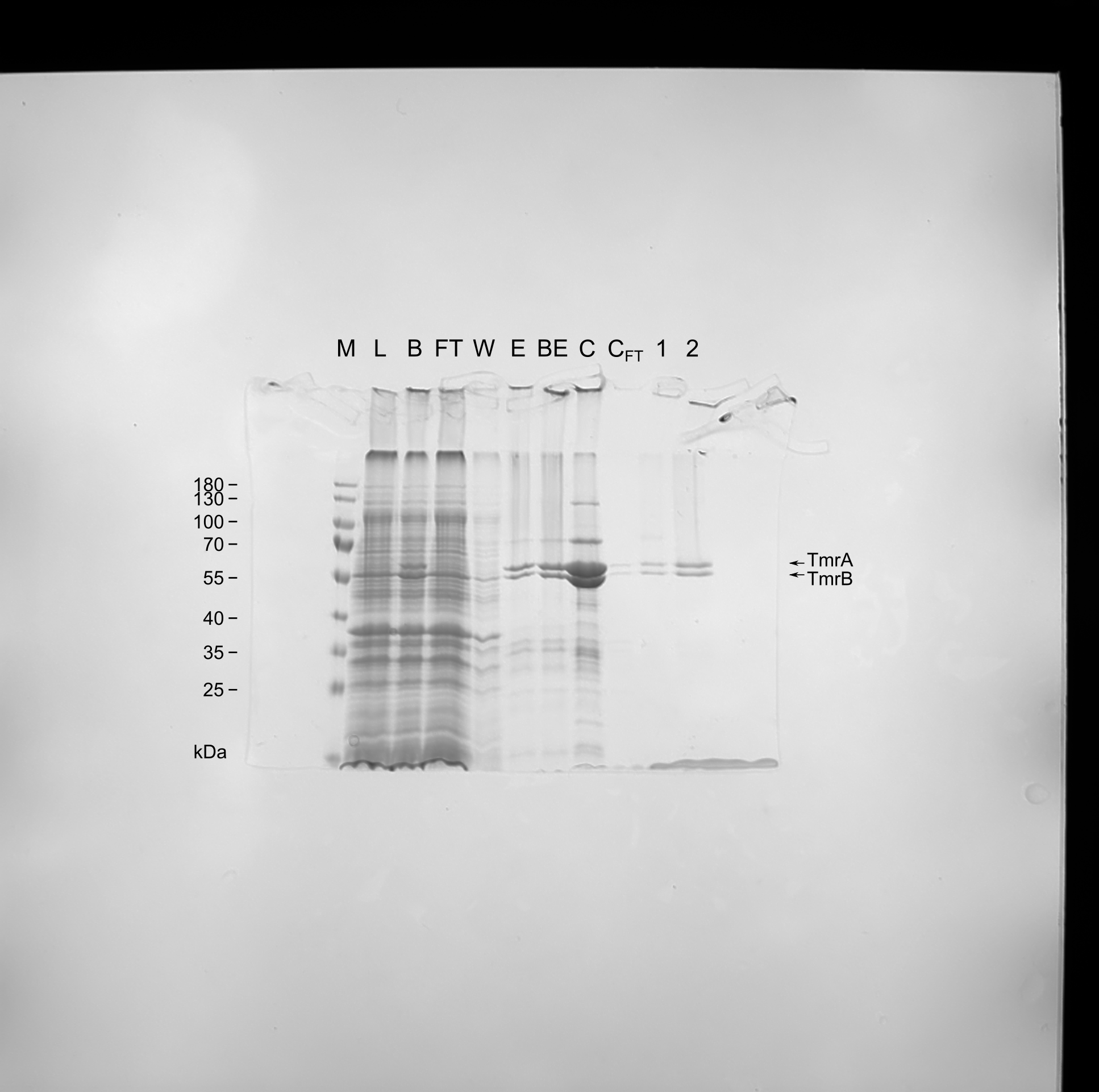

Supplement: Figure 1—figure supplement 1—source data 3. [file elife-110967-fig1-figsupp1-data3.zip › SDS-PAGE TmrAB purification_uncropped, labeled gel.png]
