# Supplementary material for: ATP-driven conformational dynamics reveal hidden intermediates in a heterodimeric ABC transporter
Source: eLife. 2026 Jul 31;15:RP110967. doi: 10.7554/eLife.110967 (PMC13427343; doi:10.7554/eLife.110967)
Supplement: Peer review history

# Editor report: eLife Assessment

**DOI:** 10.7554/eLife.110967.4.sa0

**Authors:** Camilo Perez

**Affiliations:** University of Georgia United States

**Keywords:** Convincing, Important

The study presents **important** findings revealing previously unresolved conformational dynamics of the heterodimeric type IV ABC transporter TmrAB using single-molecule FRET. The evidence presented is **convincing**, integrating careful experimental design with computational approaches to uncover states that are typically masked and difficult to detect. The work will be of interest to scientists studying the molecular mechanisms of primary active transport processes.

# Referee report: Reviewer #1 (Public review)

**DOI:** 10.7554/eLife.110967.4.sa1

Summary:

Pecak et al have deciphered the conformational dynamics of a heterodimeric model ABC transporter, TmrAB, a functional homolog of the human antigen transporter TAP, using single molecule Forster resonance energy and fluorophores attached to residues at either nucleotide binding domains or periplasmic gate. The analysis not only differentiated ATP-free and bound states, but also enabled the real time monitoring of protein conformational changes precisely dissecting transport cycles and resolving transient intermediates. This study is absolutely significant in providing and establishing a general pipeline delineating the conformational dynamics in heterodimeric ABC transporters.

Strengths:

The scientific study is very well documented for experimental design, results and conclusions supported by the experimental data. Authors have determined the conformational dynamics of TmrAB across different ATP concentrations including physiological ones and resolved an outward open state and other conformational states consistent with previous cryoEM and DEER studies. Authors have also mentioned limitations in the study.

Comments on revised version.

Authors have worked on most of the revisions stated in previous feedback and included in the newer version, which has been significantly improved. Other comments have been described to be out of scope from this study.

# Referee report: Reviewer #2 (Public review)

**DOI:** 10.7554/eLife.110967.4.sa2

In their manuscript entitled 'ATP-driven conformational dynamics reveal hidden intermediates in a heterodimeric ABC transporter', Pečak et al. use elegant single-molecule FRET experiments in detergent to investigate the heterodimeric ABC transporter TmrAB. By combining simulations of the transporter's accessible volume with elegant trapping strategies, the authors identify an unresolved outward-facing open state and conclude that it is usually obscured by a rapidly interconverting ATP-bound ensemble. Overall, the study demonstrates that smFRET can resolve the short-lived intermediate states of TmrAB and potentially other ABC transporters that are obscured in ensemble measurements.

It is a very interesting study that highlights the power of combining high-resolution structural information with spectroscopic approaches. I had three major concerns with the original version, all of which have been addressed by the authors in this revised version.

# Author comment: Author response

**DOI:** 10.7554/eLife.110967.4.sa3

**Authors:** Matija Pečak, Christoph Nocker, Robert Tampé

**Affiliations:** Goethe University Frankfurt Frankfurt Germany; Goethe University Frankfurt Frankfurt Germany; Goethe University Frankfurt Frankfurt Germany

The following is the authors’ response to the previous reviews

**Public Reviews:**

**Reviewer #1 (Public review):**

Summary:

Pecak et al have deciphered the conformational dynamics of a heterodimeric model ABC transporter, TmrAB, a functional homolog of the human antigen transporter TAP, using single molecule Forster resonance energy and fluorophores attached to residues at either nucleotide binding domains or periplasmic gate. The analysis not only differentiated ATP-free and bound states, but also enabled the real time monitoring of protein conformational changes precisely dissecting transport cycles and resolving transient intermediates. This study is absolutely significant in providing and establishing a general pipeline delineating the conformational dynamics in heterodimeric ABC transporters.

Strengths:

The scientific study is very well documented for experimental design, results and conclusions supported by the experimental data. Authors have determined the conformational dynamics of TmrAB across different ATP concentrations including physiological ones and resolved an outward open state and other conformational states consistent with previous cryoEM and DEER studies. Authors have also mentioned limitations in the study.

Comments on revised version.

Authors have worked on most of the revisions stated in previous feedback and included in the newer version, which has been significantly improved. Other comments have been described to be out of scope from this study.

**Reviewer #2 (Public review):**

In their manuscript entitled 'ATP-driven conformational dynamics reveal hidden intermediates in a heterodimeric ABC transporter', Pečak et al. use elegant single-molecule FRET experiments in detergent to investigate the heterodimeric ABC transporter TmrAB. By combining simulations of the transporter's accessible volume with elegant trapping strategies, the authors identify an unresolved outward-facing open state and conclude that it is usually obscured by a rapidly interconverting ATPbound ensemble. Overall, the study demonstrates that smFRET can resolve the short-lived intermediate states of TmrAB and potentially other ABC transporters that are obscured in ensemble measurements.

It is a very interesting study that highlights the power of combining high-resolution structural information with spectroscopic approaches. I had three major concerns with the original version, all of which have been addressed by the authors in this revised version.

**Recommendations for the authors:**

**Reviewer #2 (Recommendations for the authors):**

I mentioned that the final section of the Results part seems like an afterthought, especially since the heading suggests a broader scope.

Reply: We appreciate this comment. We have revised the final section of the Results to improve its structure and ensure that the scope indicated by the heading is fully reflected in the content. This section now more clearly integrates kinetic and thermodynamic aspects of the transport cycle.

The changes made to the section do not align with the wording of the reply. Please consider modifying it further.

We appreciate the positive feedback and this final comment. We have revised the final section of the Results to better reflect the scope indicated by the heading. In addition to clarifying the kinetic analysis, we now explicitly relate our kinetic observations to previously determined thermodynamic measurements, showing that the rapid interconversion of ATP-bound conformations observed during steady-state turnover is consistent with a thermodynamic landscape characterized by a near-zero free-energy difference and entropy–enthalpy compensation. This revision more clearly integrates the kinetic and thermodynamic aspects of the transporter cycle.
